# Classic Ehlers–Dalnos syndrome presenting as atypical chronic haematoma: a case report with novel frameshift mutation in *COL5A1*

**DOI:** 10.1186/s12887-020-02386-1

**Published:** 2020-10-27

**Authors:** Wei-Ching Chiu, Shu-Huey Chen, Mei-Chen Lo, Yung-Ting Kuo

**Affiliations:** 1 Department of Pediatrics, Shuang Ho Hospital, Ministry of Health and Welfare, Taipei Medical University, No. 291, Zhongzheng Rd., Zhonghe District, New Taipei, 23561 Taiwan; 2 Department of Pediatrics, School of Medicine, College of Medicine, Taipei Medical University, No. 250, Wu-Hsing Stree, Taipei, 110 Taiwan; 3 Taipei Cancer Center, Taipei Medical University, Taipei, Taiwan; 4 Department of Nursing, Central Taiwan University of Science and Technology, Taichung, Taiwan

**Keywords:** Ehlers-Danlos syndrome classical type, Collagen type V alpha-1 chain (COL5A1) gene, COL5A1 frameshift mutation, Collagen type V heparin-binding, Bleeding diathesis

## Abstract

**Background:**

Ehlers–Danlos syndrome (EDS) is a group of inherited connective tissue disorders characterized by skin hyperextensibility, joint hypermobility and soft tissue vulnerable to blunt injury. Early recognition and diagnosis are crucial to patients to provide appropriate treatment, as well as to screen for life-threatening conditions such as aortic dissection and hollow organ perforation. The diagnosis of EDS is made based on clinical presentations, skin biopsy, and electron microscopy findings. To date, mutations in at least 20 genes have been found to cause the Ehlers-Danlos syndromes. However, EDS is still underestimated due to lack of awareness of its variable clinical presentations. Here we reported an EDS case with atypical initial presentation and a novel genetic mutation.

**Case presentation:**

This 4-year-old Taiwanese male patient presented with easy bruising, multiple ecchymoses, joint hypermobility, hyperextensible skin, and prolonged pretibial haematoma. He was initially suspected of a bleeding tendency due to coagulation disorders. The coagulation test results were normal. DNA sequencing was performed for molecular diagnosis. Subsequently, the diagnosis of classical EDS was made by identifying a novel frameshift mutation in *COL5A1* [NM_000093.4:c.4211_4212delAG, p.Gln1404Arg]. This mutation in the type V collagen gene *COL5A1* contributes to the phenotype of classical EDS. This novel frameshift mutation may disturb the structural stability of collagen V and interfere with its heparin binding capacity, explaining the chronic haematoma.

**Conclusion:**

The reported case showed the unusual features of chronic haematoma. This novel frameshift mutation and its phenotype correlation can provide useful information for practitioners about early recognition in Ehlers–Danlos syndrome.

## Background

Ehlers–Danlos syndrome (EDS) is a broad-spectrum hereditable disorder of connective tissue. In patients with EDS, accurate diagnosis is crucial to screen for life-threatening complications, such as vascular and hollow organ perforation, ligamentous laxity, and joint subluxation that can lead subsequent pain and long-term disability [1]. EDS was once recognized as a rare disease with the prevalence of all forms of EDS was previously estimated at 1:150,000 in the previous studies [2]. In the United Kingdom, a national electronic cohort study suggested a higher prevalence was estimated at 194.2 per 100,000 in 2016/2017 or roughly 10 cases in 5000 patients [3]. These prevalence differences can arise from poor recognition in children and consideration of initial symptoms as normal and irrelevant episode. Based on the latest 2017 international classification of EDS, there are 13 subtypes of EDS that are identified based on clinical characteristics, inheritance pattern, and molecular characteristics: classical, classical-like, cardiac-valvular, vascular, hypermobile, arthrochalasia, dermatosparaxis, kyphoscoliotic, Brittle cornea syndrome, spondylodysplastic, musculocontractural, myopathic, and periodontal [4]. Classical EDS (cEDS) is autosomal dominant genetic disorder, and cEDS together with the hypermobility type accounts for approximately 90% of all cases of EDS. It arises due to mutations in type V collagen genes (*COL5A1* and *COL5A2*) and is characterized by marked joint hypermobility, hyperextensible skin, hemosiderotic scars, and cutaneous pseudotumors [5]. A definite diagnosis can be made by recognizing a pathognomonic mutation in *COL5A1* or *COL5A2*. However, their symptoms overlap between various types of EDS and other hereditary connective tissue disorders including osteogenesis imperfecta and Marfan syndrome, thus making the timely diagnosis of EDS challenging. Many cases are undiagnosed or misdiagnose, and delayed diagnosis will cause impedance of appropriate treatment and preventive strategies [3, 6]. Here, we report a Taiwanese patient who was diagnosed with cEDS and who harbored a novel frameshift mutation in *COL5A1* [NM_000093.4:c.4211_4212delAG, p.Gln1404Arg] that was detected on Sanger sequencing.

## Case presentation

**Fig. 1:**
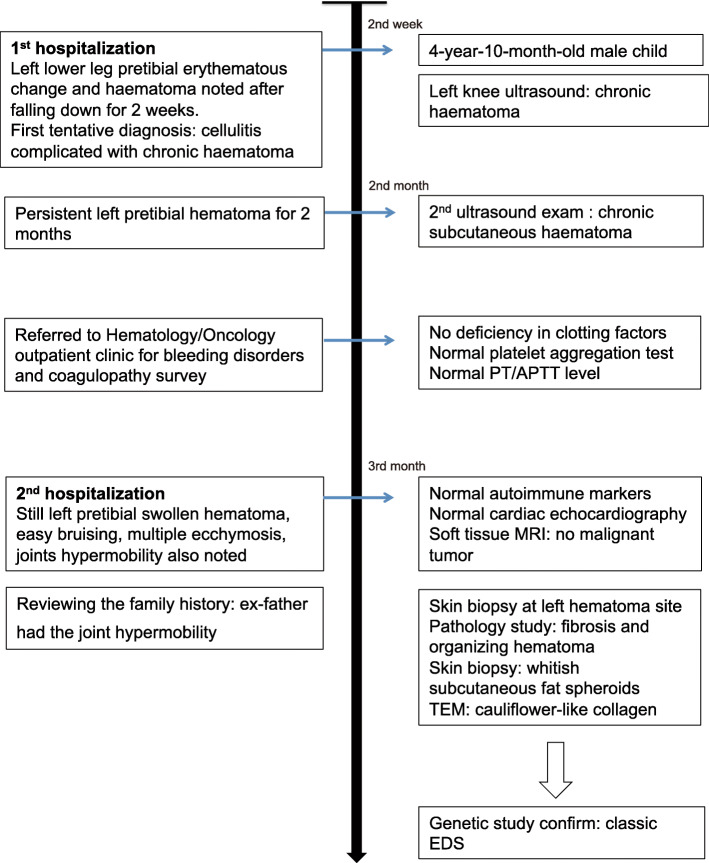
Timeline of clinical course (legend: PT: prothrombin time, APTT: activated partial thromboplastin time, TEM: Transmission electron microscopy)

We presented the case of a 5-year-1-month-old boy who was referred to our outpatient clinic when he was 4-year-10-month-old (timeline of clinical course—Fig. 1 illustrates the clinical course).

**Fig. 2:**
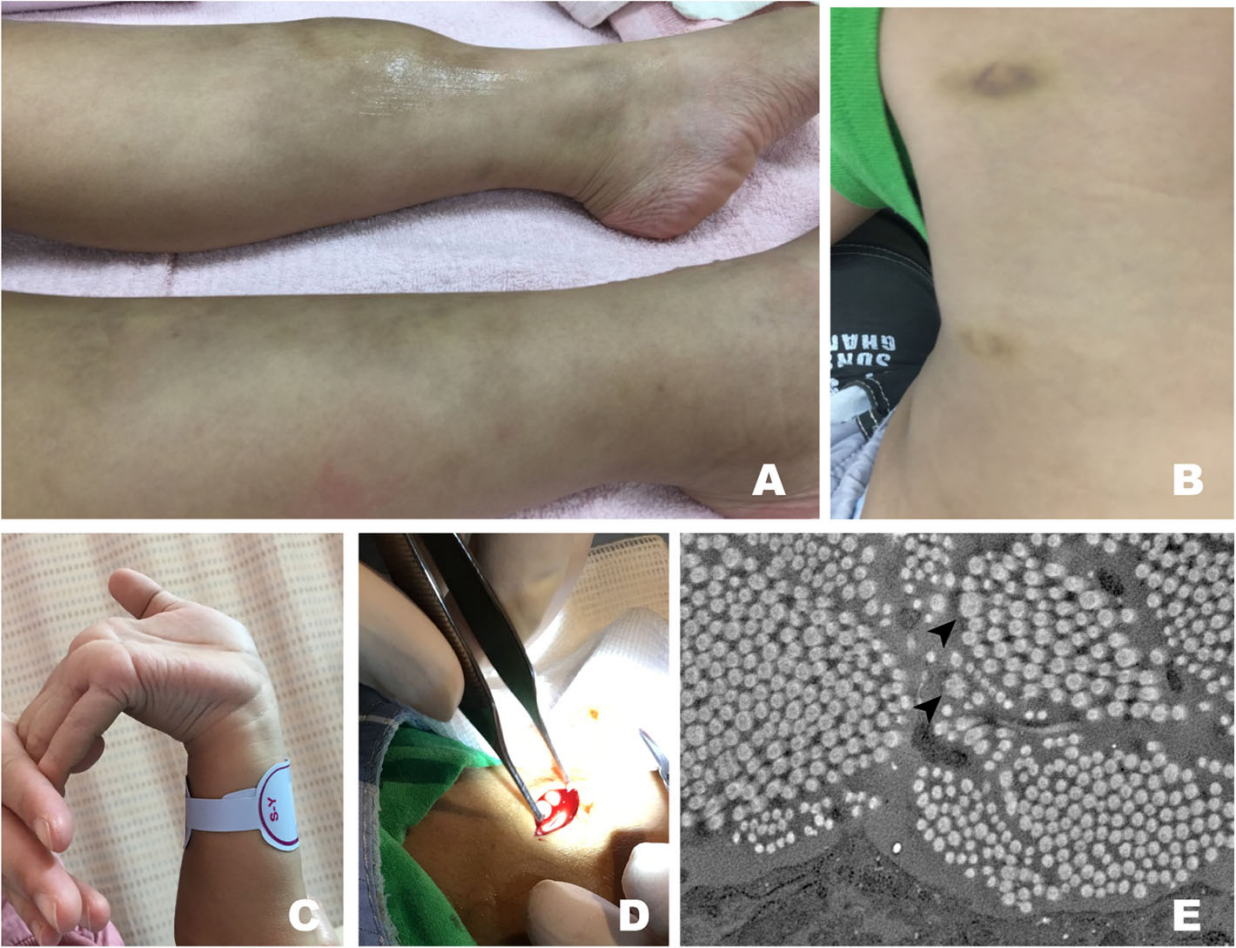
Clinical presentations and electron microscopic examination. **a**. Chronic haematoma in the lower pretibial area. **b**. Multiple bruising ecchymosis on the anterior upper chest and flank areas. **c**. Hypermobility of the wrist joint and hyperextension of the proximal digital joints. **d**. Subcutaneous fat spheroids observed in the skin biopsy. **e**. “Collagen followers” (closed black arrow, ➤) in electron microscopic examination

**Fig. 3:**
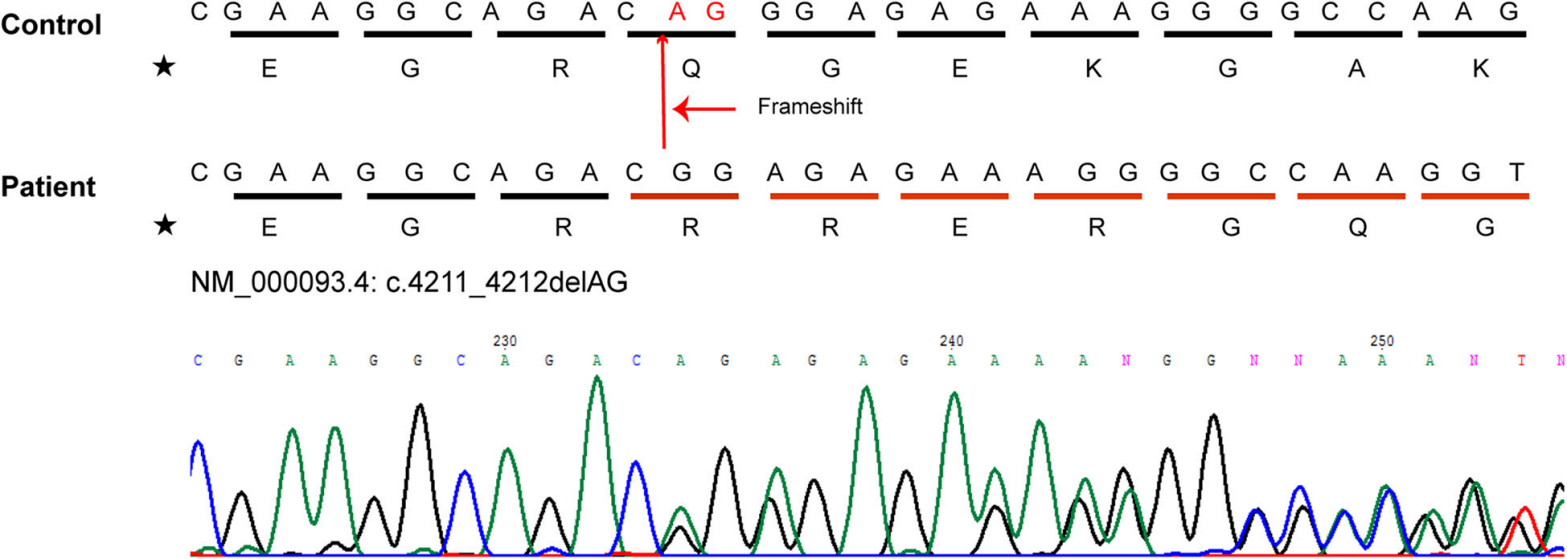
Genetic sequence of *COL5A1* mutation. Genetic studies revealed a novel frameshift mutation in exon 54. This mutation replaces glutamine (Q) with arginine (R) and the following amino acid codons. ★, amino acids are represented by the letters used in the international nomenclature

The patient was examined because of prolonged unresolved pretibial haematoma (Fig. 2a) that measured 6.5 × 6.5 cm^2^ after falling down to the floor with initial impression of cellulitis. Easy bruising, multiple ecchymoses (Fig. 2b), joint hypermobility (Fig. 2c) and poly-arthralgia were noted since childhood; however, no specific medical advice was sought before the clinic visit. Clinical examination revealed body height 108 cm (23th percentile) and body weight 21 kg (75th percentile). The patient had joint hypermobility, Beighton score 7 (including bilaterally passive dorsiflexion of fifth finger beyond 90, score 2; bilaterally passive flexion of thumb to forearm, score 2; hyperextension of the elbow beyond 10 degree, score 0; bilateral hyperextension of the knee beyond 10 degree, score 2; forward flexion of the trunk with knees fully extended and palms resting on the floor, score 1) [7], poly-arthralgia without inflammatory signs, mildly hyperextensible skin, and ecchymoses over multiple sites. Among the minor diagnostic criteria for cEDS, muscle hypotonia, delayed gross motor development, easy bruising without obvious causes, and positive family history (similar symptoms of joint hypermobility in his father and aunt) were also observed. Chest wall deformity, kyphoscoliotic posture, periodontal lesions were not observed during physical examination. No tumors and vasculopathy were observed on ultrasound and magnetic resonance imaging of the pretibial mass. Echocardiographic assessment revealed no mitral valve prolapse and aortic root dilatation. The myopathy was less likely due to normal range creatine kinase. Hematological studies for coagulation and platelet disorders and study for autoimmune diseases all provided normal results (Supplementary Table. 1). With aforementioned presentation, the patient was suspected of having EDS. Skin biopsy was performed, and whitish subcutaneous fat spheroids were observed during the biopsy (Fig. 2d). Light microscopy revealed fibrosis in the subcutaneous fat and organizing haematoma without malignancy. Transmission electron microscopy of the skin biopsy revealed irregular interfibrillar spaces, collagen fibers with variable diameter, and collagen cauliflowers (Fig. 2e) [8]. Molecular evaluation of genomic DNA extracted from a blood sample was performed. Whole exome sequencing and conventional Sanger sequencing were performed using the CytoOnearray sequencing panel (Phalanx Biotech, Taiwan). These analyses identified the frameshift mutation NM_000093.4(COL5A1):c.4211_4212delAG in exon 54, which resulted in a glutamine to arginine substitution at codon 1404 [NP_000084.3: p.Gln1404ArgfsTer77] (Fig. 3). Based on disease-causing mutation databases, such as Leiden Open Variation Database (LOVD) [9] and ClinVar [10], this frameshift mutation identified in our patient is a novel finding. At least 45 pathologic variants of a frameshift mutation in cEDS have been found; however, no pathological variants and mutation loci were detected, as in our patient. This is a novel frameshift mutation in *CO5A1* first identified in this patient with cEDS. This patient was regularly followed in the multidisciplinary team and accepted the rehabilitation program in outpatient clinic.

## Discussion and conclusion

### Considering chronic haematoma as another disease entity

Unreasonable ecchymoses and increased bleeding diathesis are crucial clinical characteristics of several coagulation and platelet disorders, such as von Willebrand disease and hemophilia A and B. However, these characteristics can also be observed in hereditary collagen disorders, which belong to a heterogeneous group of genetic diseases that are caused by mutations in structural collagen genes or in genes that code for enzymes involved in their post-translational modifications. EDS may be regarded as one of the differential diagnoses in such cases [11].

### COL5A1mutations involved in cEDS

The main etiology of cEDS involves structural defects in type V collagen. Type V collagen is widely distributed in tissues, and it is a quantitatively minor fibrillar collagen. Together with type I collagen, it plays a main role in collagen fibrillogenesis [12]. The commonest isoform of type V collagen is a heterotrimer composed of two α1-propeptide chains and one α2-propeptide chain encoded by *COL5A1* and *COL5A2*, respectively. The α1-propeptide chain is composed of three domains: N-propeptide, α-helix, and C-propeptide [13]. *COL5A1* cDNA comprises 66 exons distributed over more than 150 kb of genomic DNA. The most common null mutations detected in *COL5A1* consist of frameshift, nonsense, and splice site mutations that result in a premature termination codon (PTC) [14, 15], which generates unstable mutant *COL5A1* mRNA that is rapidly degraded, resulting in the overall reduction of type V collagen [15]. In approximately 40–50% of individuals with cEDS, nonsense or frameshift variants are responsible for a nonfunctional *COL5A1* allele [16]. *COL5A1* haploinsufficiency mutations are the predominant mutations, and they result in the reduction of type V collagen, whereas a minority of mutations result in the production of structurally abnormal type V collagen [17].

### Disruption of type V collagen and heparin binding site interaction

Type V collagen binds to DNA, heparan sulfate, thrombospondin, heparin, and insulin. Previous studies have reported the structural requirements and molecular mechanisms of the interactions of heparin and heparan sulfate with collagen V [18–20]. A fragment (Ile824-Pro950) of the collagen 1(V) chain, called HepV, binds to heparin through a cluster of three major basic residues—Arg912, Arg918, and Arg921—and two additional residues—Lys905 and Arg909 [21]. Triple-helical molecules with the homotrimeric or heterotrimeric collagen V molecules form very stable complexes with heparin and heparan sulfate [20]. Structural disability of collagen V interferes with heparin binding, resulting in dysfunction of cell–matrix interactions and contributing to the molecular pathogenesis of cEDS.

In our case, the patient demonstrated the characteristics of easy bruising and multiple ecchymoses in addition to skin fragility, hyperextensibility, and joint hypermobility. Skin biopsy revealed subcutaneous spheroids due to herniation of the subcutaneous fat; these spheroids were small, hard, and cyst-like nodules. With fragile collagen matrix protein in the surrounding tissue, these fat spheroids are freely movable over the bony protuberances along the extremities. The dermis in our patient contained large collagen fibrils of irregular diameters (collagen “cauliflowers”), which are indicative of disturbed collagen fibrillogenesis [6]. Genetic studies have revealed the frameshift mutation c.4211_4212delAG in exon 54, which resulted in the replacement of glutamine with arginine at position 1404 [p.(Gln1404Arg)]. Arginine is one of the destabilizing residues observed in the Gly position of the Gly-X-Y pattern that is repeated in the collagen triple helix [22, 23]. Collagen represents approximately 40% of the total protein in a vessel wall, and type V collagen is one of the widely distributed types of collagen in vessels. Collagen is the only matrix protein that supports both platelet adhesion and complete activation, and it has an imperative role in initiating homeostasis. If the collagen structure is abnormal, the vessel wall is more vulnerable to injury, and the homeostasis function is altered. This frameshift mutation detected in our patient may explain the phenomena of chronic haematoma, multiple ecchymoses, and easy bruising, which are due to the disturbance of the structural integrity of the triple helix and interference with its heparin-binding capacity.

## Conclusions

This reported case showed initial symptoms with chronic pretibial haematoma. We identified a novel frameshift mutation in *COL5A1* in a patient with cEDS. This mutation disturbed the structural stability of collagen V and interfered with its heparin-binding capacity; this is one of the molecular mechanisms contributing to cEDS. The patient’s clinical symptoms, skin biopsy, in combination with molecular test can help practitioners increase the awareness of this disease entity and take stepwise diagnostic approaches to screen risky complications.

## Supplementary information

Additional file 1: **Table S1.** Crucial laboratory findings. (TIF 58 kb)

Additional file 2. (TIF 103 kb)

## Data Availability

The datasets used and analyzed during the current report are included in this published article and its supplementary information files.

## Abbreviations

EDS: Ehlers-Danlos syndrome
cEDS: Classic Ehlers-Danlos syndrome
COL5A1: Collagen Type V Alpha 1 Chain
COL5A2: Collagen Type V Alpha 2 Chain
DNA: Deoxyribonucleic acid
PTC: Premature termination codon
