# Supplementary figures and images for: Classic Ehlers–Dalnos syndrome presenting as atypical chronic haematoma: a case report with novel frameshift mutation in COL5A1
Source: BMC Pediatr. 2020 Oct 27;20:495. doi: 10.1186/s12887-020-02386-1 (PMC7590603; doi:10.1186/s12887-020-02386-1)

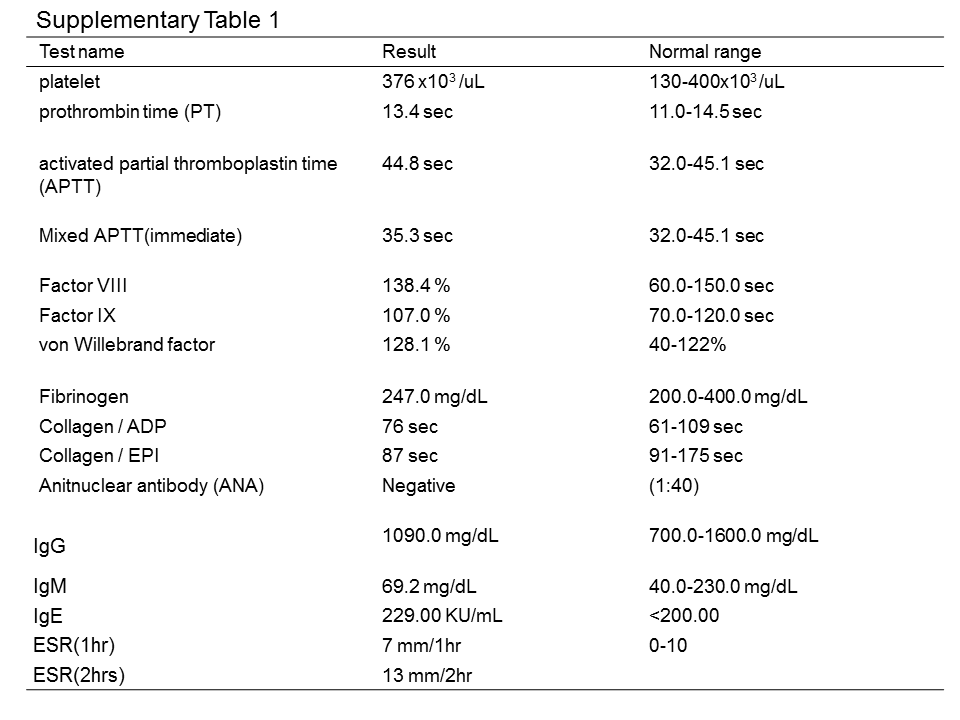

Supplement: Supplementary file 1 — Table S1. Crucial laboratory findings. (TIF 58 kb) [file 12887_2020_2386_MOESM1_ESM.tif]

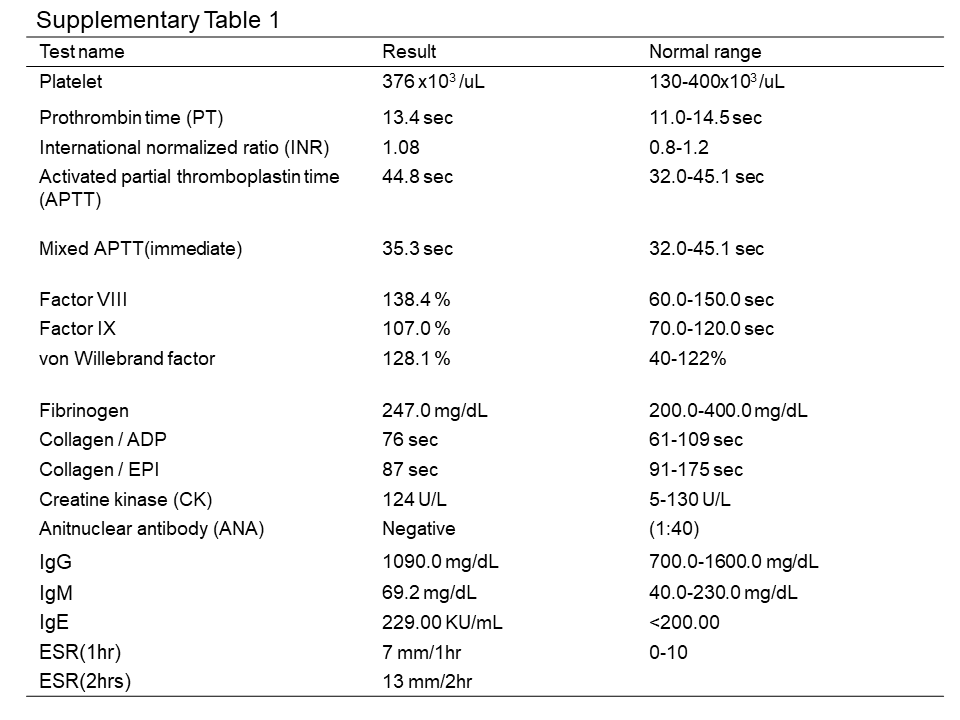

Supplement: Supplementary file 2 — (TIF 103 kb) [file 12887_2020_2386_MOESM2_ESM.tif]
